# Improved Field Emission Properties of Carbon Nanostructures by Laser Surface Engineering

**DOI:** 10.3390/nano10101931

**Published:** 2020-09-27

**Authors:** Minh Nhat Dang, Minh Dang Nguyen, Nguyen Khac Hiep, Phan Ngoc Hong, In Hyung Baek, Nguyen Tuan Hong

**Affiliations:** 1 The Australian Research Council (ARC) Industrial Transformation Training Centre in Surface Engineering for Advanced Materials (SEAM), Faculty of Science, Engineering and Technology, Swinburne University of Technology, P.O. Box 218, Hawthorn, VIC 3122, Australia; 2 Department of Chemistry, University of Houston, Houston, TX 77204-5003, USA; dangminh27498@gmail.com; 3 Vietnam Academy of Science and Technology (VAST), University of Science and Technology of Hanoi, 18 Hoang Quoc Viet, Hanoi 100000, Vietnam; 4 Centre for High Technology Development, VAST, 18 Hoang Quoc Viet, Hanoi 100000, Vietnam; hiephulk@gmail.com (N.K.H.); hongpn@htd.vast.vn (P.N.H.); 5 Korea Atomic Energy Research Institute, Daeduk-Daero 989-111, Daejeon, Korea; ihbaek@kaeri.re.kr

**Keywords:** carbon nanotubes, hot-filament CVD, graphene, field electron emission, laser machining

## Abstract

We herein present an alternative geometry of nanostructured carbon cathode capable of obtaining a low turn-on field, and both stable and high current densities. This cathode geometry consisted of a micro-hollow array on planar carbon nanostructures engineered by femtosecond laser. The micro-hollow geometry provides a larger edge area for achieving a lower turn-on field of 0.70 V/µm, a sustainable current of approximately 2 mA (about 112 mA/cm^2^) at an applied field of less than 2 V/µm. The electric field in the vicinity of the hollow array (rim edge) is enhanced due to the edge effect, that is key to improving field emission performance. The edge effect of the micro-hollow cathode is confirmed by numerical calculation. This new type of nanostructured carbon cathode geometry can be promisingly applied for high intensity and compact electron sources.

## 1. Introduction

Electron sources have been utilized in X-ray tubes, vacuum microwave amplifiers, and electron guns [1]. In these instruments, thermionic emitters are widely used due to their proven operation. However, the thermionic emitters are bulky and not efficient enough for low-power applications due to the waste heat radiated from the filament and the high applied field. An alternate means to generate electrons is field emission (FE), which exhibits many advantages in comparison with thermionic emission. For instance, as field emitters do not require heat to generate electrons, they are more energy-efficient and may be rapidly switched on and off. Among the family of field emission materials, carbon nanostructures are interesting due to low applied fields and elevated electron currents [2].

Electron field emission from the nanostructured carbon is found to involve the phases showing sp^2^ hybridization. The sp^2^ content, orientation, and size of these phases, which have a positive electron affinity, can play a considerable role in emissions at a low field [3,4]. Graphene and carbon nanotubes are the types of cold cathodes that extract electrons at the lowest applied field.

Low applied fields of the nanostructured carbon cathodes are also facilitated by the large aspect ratio. For nearly two decades, graphene has drawn significant attention from scholars owing to its extraordinary properties, which are applicable in diverse industries such as aerospace, energy, and infrastructure construction [5,6,7]. Graphene and its relative composites can be synthesized via bottom-up and top-down methodologies including chemical vapor deposition (CVD), liquid exfoliation, and electrochemical co-synthesis [8,9,10,11,12]. For graphene materials, the existence of abundant sharp-edged structures can act as efficient field emission sites [13,14]. Nevertheless, field emission from graphene is still a challenge because the fabrication methods such as exfoliation or screen-printing will create graphene sheets laterally on the support substrate, which is unfavorable for electron tunneling.

Carbon nanotubes (CNTs) are another novel carbon allotrope, which can be seen as cylindrical graphene sheets [15]. Because of their long, tubular geometry and excellent physicochemical properties, carbon nanotubes are also attractive materials for cold cathodes [1,2,3]. Planar and point structures are the two predominant types of carbon nanotube cathodes. The CNT point emitter is favorable in devices requiring a small, bright electron source [16], whereas the CNT planar emitter is suited to applications requiring uniform and relatively large emitting sites. Such applications widely use dense carbon nanotubes as the cathode. The dense carbon nanotubes are deposited on the substrate by either CVD, or printing [17]. CVD carbon nanotubes are selectively chosen for devices requiring low turn-on field and high emitting current. Individual carbon nanotubes demonstrate excellent field emission properties; however, vertically-aligned carbon nanotubes (VACNTs) do not present such great performance. In this report, the VACNTs are defined as dense carbon nanotubes grown perpendicular to the substrate platform. This disadvantage is acceptably ascribed to the screen effect, which is mutual shielding of the electric field between adjacent nanotubes [18]. Some studies have reported mitigation of the screen effect by photolithography patterning [19]. One can obtain the high aspect-ratio geometry of patterned VACNTs purposely, which is favorable to local electric field enhancement. The patterned VACNTs such as VACNT-column arrays could not only negate the screen effect but also introduce isolated CNT extrusions and edges, both of which result in the enhanced electric field [20]. 

Surface engineering methods are known to enhance the pristine properties of bulk materials [21,22,23,24]. For cold cathodes, geometry engineering and surface modification are alternative methods to improve field emission performance [25,26,27]^.^ In this work, we use a femtosecond laser to engineer and hence enhance the electric field of the VACNT cathode. The femtosecond laser is used to selectively trim out carbon nanotubes and create a micro-hollow (MH) array on the top surface. Since the MH-VACNTs cathode is based on the as-grown VACNTs, our work included the pristine and planar VACNTs to compare their field emission properties. The field emission characteristics of the MH-VACNTs cathode showed a low turn-on field, and both stable and high emission current. Initial results demonstrated the 40 μm diameter hollow cathodes with a pitch of 150 μm can achieve a turn-on field of 0.70 V/μm, lower than the 0.89 V/μm of the planar VANCTs (without laser modification). Numerical calculation confirmed the local electric field is increased at the rim edge of the micro hollow, a key factor to improving field emission characteristics.

## 2. Experimental Procedure

### 2.1. Materials Preparation

The MH-VACNTs cathode was produced by a two-step process consisting of (i) the VACNTs synthesis and (ii) the femtosecond laser engineering. The VACNTs were grown on silicon (Si) by using the hot-filament CVD method [20,28]. The highly doped, 500 μm thick silicon substrate, following the microelectronic industry standard, was used for the growth of carbon nanotubes. The catalysts are selectively deposited using a routine photolithography process. In brief, the VACNT film was obtained at 750 °C, CH_4_/H_2_ mixture (20/30 SCCM-standard cubic centimetre per minute at Standard Temperature and Pressure-STP), and reactor pressure of 30 Torr. It is noted that the hot-filament CVD has the advantage of decomposing CH_4_/H_2_ at relatively high temperatures (2200–2500 °C), which is favourable to a high purity of the as-grown carbon nanotubes [28].

The 1.5 mm diameter VACNTs with a thickness of 250–300 μm was subject to femtosecond laser machining in an ambient atmosphere. A femtosecond laser is a tool that is widely used to fabricate microstructures from several materials, including carbon nanotubes [29]. The laser system consisted of a mode-locked Ti:Sapphire laser (repetition rate of 92 MHz, 80-fs pulse width, average laser power of 200 mW) and the translation stage [30]. Once the laser beam was incident on the sample, it selectively ablates carbon nanotubes. By translating the VACNT substrate stage relative to the laser shots, a periodic array of the micro hollows is formed. Using good selection of laser power, exposure time, and depth of focus, we can achieve the well-shaped hollow geometry on the VACNT film. 

### 2.2. Characterization Preparation

Carbon nanotubes were examined by scanning electron microscope SEM (FESEM S-4800, Hitachi, Tokyo, Japan) and Raman spectroscope (IFS/66 Bruker system, Billerica, MA, USA). Raman signal was recorded at room temperature using an excitation laser wavelength of 1064 nm. The 1.5-millimetre-diameter and about 250-µm-thick VACNTs on Si with and without the laser engineering are field emission (FE) tested. The FE measurement is carried out in a vacuum chamber (KVM-T4060 system, Korea Vacuum, Daegu, Korea) with a working pressure of 5 × 10^−6^ Torr and a ballast resistor of 200 kΩ. Keithley-248 (Tektronix, Beaverton, OR, USA) is a DC anode voltage source. The emission electron current is measured by Keithley-2001 (Multi-meter, Tektronix, USA). Measured FE data are acquired using LABVIEW software and a personal computer through a general-purpose interface bus (GPIB) card. The diode configuration is used with the anode (aluminium plate) and the carbon nanotube cathode. The anode and the cathode were electrically isolated by Kapton sheets (spacer); the electric field was calculated to be the anode voltage divided by the anode-to-CNT-surface distance. Current densities were estimated from the net currents and the VACNT area. Turn-on field and threshold field are defined as the electric field necessary to extract 100-nA emission current and 1-mA/cm^2^ current density, respectively. For current-voltage (I-V) curves, measurements were repeated several times until the curve became stable. The carbon nanotube cathode was also tested in a continuous operating condition for a stability test. The operation stability was evaluated by emission current values at anode voltages with a 50% duty cycle. In practice, each cycle is 60 s; hence, the anode voltage was ON for only 30 s.

To investigate the carbon nanotube cathode geometry, we carried out numerical calculation (COMSOL Multiphysics, 3D simulation) in a diode configuration. Due to periodicity in geometry and hence, electric field, a diode with a 3 × 3 micro-hollow array was used as a stand in for the whole micro-hollow array cathode. A cathode structure that modeled a practical MH-VACNTs (40 μm diameter hollow, depth of about 200 μm) is simulated. The electric field is computed by the Laplace equation solution using finite element analysis (a bias voltage of 3000 V, equivalent to an applied field of 2.40 V/μm). Pitch *d* (hollow-to-hollow distance) is a variant in the simulation. The local electric field at a distance immediately above the cathode was calculated and studied. 

## 3. Results and Discussions

### 3.1. Characterization Data

**Figure 1:**
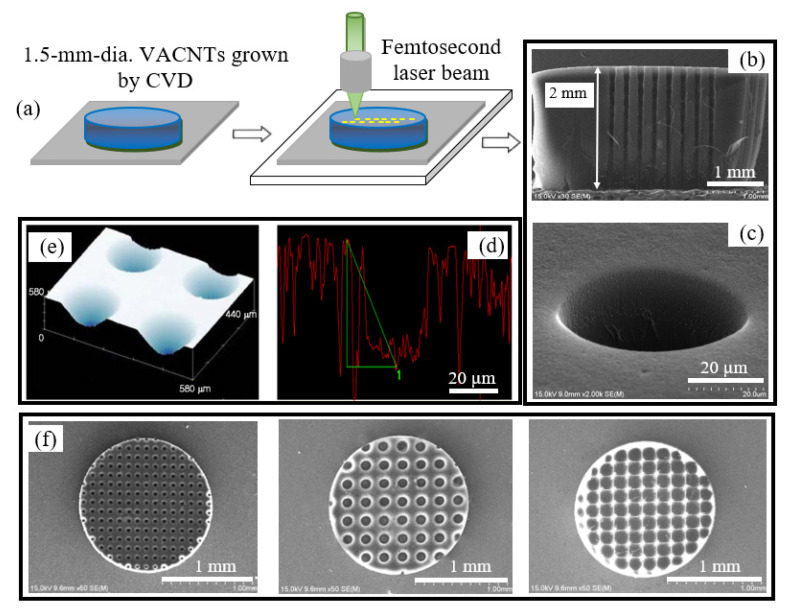
(**a**) Sketch of femtosecond laser process (repetition rate of 92 MHz, 80-fs pulse width, an average power of 200 mW); (**b**,**c**) SEM images of about 2-mm depth hollow cross-section (from the VACNT top to the bottom substrate and hollow shape on the top surface; (**d**,**e**) 3D confocal image and profile of the micro hollow with depth of ~200 µm, (**f**) typical micro-hollow arrays on 1.5 mm diameter, VACNT specimens, with depth of ~200 µm, and hollow diameters of 40, 100, and 150 µm, respectively (from left to right).

Figure 1a demonstrates the femtosecond-laser process to modify the VACNTs and the obtained results. In practice, the femtosecond laser beam is synchronized with the substrate translation, capable of creating a periodic micro-hollow array. Figure 1b,c presents typical SEM images of the micro hollow on the surface and along the VACNT bulk. The micro hollows were well-shaped and smooth. The femtosecond laser process was able to form a long micro-hollow from the VACNT top to the bottom substrate. A confocal microscope was used to draw the hollow profile and measure the depth (Figure 1d,e). Figure 1f shows typical micro-hollow arrays on the 1.5 mm diameter specimens; the hollow diameter of 40–150 µm and depth of about 200 µm were controlled by periods of the laser exposure time.

**Figure 2:**
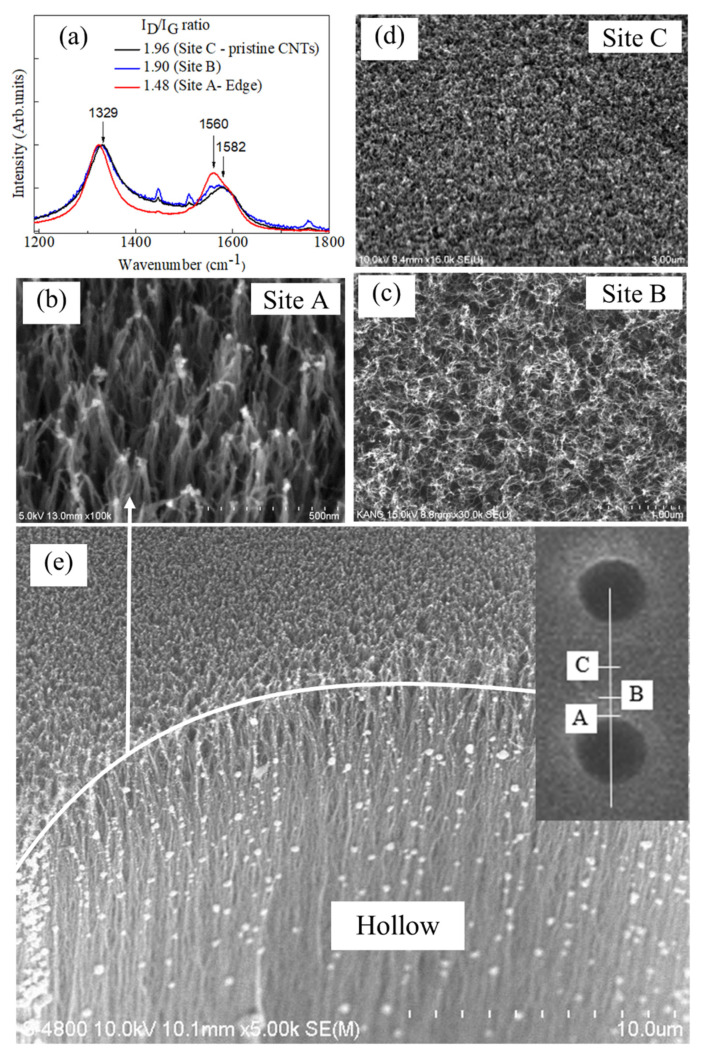
(**a**) Raman spectra of carbon nanotubes recorded at three different sites (**A**–**C**), which are shown in SEM images (**b**–**d**); (**e**) Hollow edge of CNTs, Inset: Site A, B, and C positions: (**A**) rim edge of hollow; (**B**) 25 μm away from site A; (**C**) middle between two consectutive hollows.

Raman spectra of carbon nanotubes in this work are presented in Figure 2a. Raman signals were directly recorded from the MH-VACNT (hollow diameter of 40 µm, pitch *d* ~ 100 µm) cathode at three spots, named site A, B, and C. Site A was on the rim edge, site B was 25 µm away the edge, and site C was a middle spot between two consecutive hollows. In practice, it was found that the Raman spectrum of carbon nanotubes at site C was comparable to those of the as-grown, pristine carbon nanotubes; we assumed those nanotubes (site C) were insignificantly affected by the femtosecond laser process. In Figure 2a, there occurs a graphitic G band at 1560–1580 cm^–1^ and a defective D band at ~1324 cm^–1^ [31]. The disordered peak (D-band) accounts for amorphous carbon, contaminants, and defective nanotubes as well. It is widely accepted that the intensity ratio of I_D_ to I_G_ (I_D_/I_G_) is an indicator of carbon nanotube crystallinity [32]. The I_D_/I_G_ ratio at sites A, B, and C (shown in Figure 2b–d) are 1.48, 1.90, and 1.96, respectively. The G-band of type-A CNTs increases due to the graphene zone-folding effect at the rim edge. Because of the nature of our rim-edge CNTs, which possibly have active phonon coupling to the continuum of electronic states, the red-shift of the optical phonon is associated with the increase in the temperature due to the combinational effect of thermal expansion and temperature contribution, which resulted in the finer crystal lattice of type-A CNTs. Apparently, when the temperature increases at the spot femtosecond laser hit, the G-band is also noticeably broader together with red-shifting. The nanotubes at the rim edge were also observed to be joined together as multi-junctions after the laser process (Figure 2b) which was previously reported [29,33]. Conversely, carbon nanotubes at site B may be less affected by the femtosecond laser energy; hence, their I_D_/I_G_ ratio was insignificantly reduced and mostly comparable with the pristine carbon nanotubes. However, their morphologies were more or less restructured, as can be witnessed in Figure 2c.

### 3.2. Field Emission Properties

**Figure 3:**
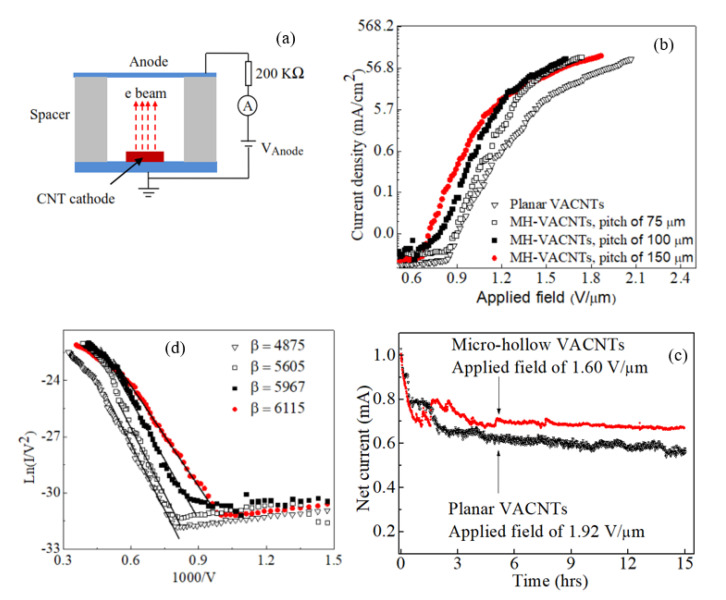
(**a**) Schematic of the field emission setup; (**b**) Current density–applied field (J–F) curves of 1.5 mm dia. MH-VACNTs (40-µm-dia. hollow, depth of ~ 200 µm) with pitch of 75, 100, and 150 µm, and planar VACNTs; (**c**) Stability test of two cathodes: MH-VACNTs (40-µm-dia. hollow, depth of ~200 µm, and pitch of 150 µm) and planar VACNTs, constant bias voltage, 50% duty cycle; (**d**) Corresponding F-N plots.

To examine the operation of both the MH-VACNTs and planar VACNTs cathodes, field emission characteristics were repeatedly measured. The MH-VACNTs, the same 40-µm-dia. hollow, depth of ~200 µm, with pitch of 75, 100, and 150 µm, respectively were measured. The measuring setup is demonstrated in Figure 3a. The anode–cathode spacing was maintained at 1500 µm by a Kapton spacer. The current density–applied field (J–F) curves of both cathodes are shown in Figure 3b. The turn-on field, corresponding to an emission current of 0.1 μA, was 0.70 V/μm and 0.89 V/μm for the MH-VACNTs (40-µm-dia. hollow, depth of ~200 µm, and pitch of 150 µm) and the planar VACNTs, respectively. We extended further field emission measurements to a higher current range by gradually increasing applied fields. The current density reached 10 mA/cm^2^ at the threshold field of less than 1.50 V/μm (the MH-VACNTs cathodes). The stability of field emission current was also measured. Before the stability test, an aging process was carried out. Anode voltages of 100–3000 V were swept up/down several times. The aging experiment is aimed to exercise field emission of carbon nanotubes in a wide range of applied fields. The experiment may also remove loosely-entangled carbon nanotubes due to laser machining. The stability test of both cathodes was started at an emitting current of 1 mA. Figure 3c demonstrates emitting current over 15 h. The constant bias voltage, 50% duty cycle, equivalent to an electric field of 1.60 V/μm, and 1.92 V/μm for the MH-VACNTs (40-µm-dia. hollow, depth of ~200 µm, and pitch of 150 µm) and the planar VACNTs, was applied respectively. Although the electrical aging has been carried out, in the onset both cathodes showed a gradual decrease and large fluctuation of the emission current before the stable electron emission was established. It was widely accepted that field emission is a highly selective process, which occurs at a relatively small number of emitting sites with the largest field enhancement factor *β*. Due to localized high currents, the best-first emitting sites may fail first or collapse abruptly, and the emitting currents are sharply decreased. Then, many other sites that have a more average field enhancement factor start to emit electrons. Because of large numbers and even distribution, these new emitting sites operate reliably, and hence, total electron currents become more stable. For the MH-VACNTs, the standard deviation of the observed emitting currents within two hours (from fifth hour) was about 5.15 μA (the mean current value of 700 μA) and smaller than that of the planar VACNTs (10.5 μA with the mean current value of 615 μA). Therefore, the MH-VACNT cathode operated in a more reproducible manner than the planar VACNTs.

Generally, electron emission of carbon nanotubes, driven by quantum mechanical tunneling, follows the simplified Folwer–Nordheim (F-N) equation [34]

(1) $$I=A\;\frac{1.42\times 10^{-6}}{\phi }\;F^{2}\;\exp\left(\frac{10.4}{\sqrt{\phi }}\right)\;\exp\left(-\frac{B\;\phi^{1.5}}{F}\right)$$

where *A* is the emitting area (m^2^), *B =* 6.44 × 10^9^ VeV^–1.5^m^–1^ (constant); *φ* is work function (*eV)*, and *F* represents local electric field. The local electric field *F* is estimated approximately by *F* = $\beta \frac{V}{d}$, in which *V* is the applied bias voltage, *d* is the anode to CNT-top distance, and *β* is the collective field enhancement factor. Electron emission at low applied fields is possible because of the significant amplification at of the local field. Therefore, *β* depends on the distribution density and geometry of the carbon nanotube cathode. F–N equation can be simplified as

(2) $$I=A\;\frac{1.42\times 10^{-6}}{\phi }\;\beta^{2}\;{\left(\frac{V}{d}\right)}^{2}\exp\left(\frac{10.4}{\sqrt{\phi }}\right)\;\exp\left(-\frac{B\;\phi^{1.5}d}{\beta V}\right)$$

**Figure 4:**
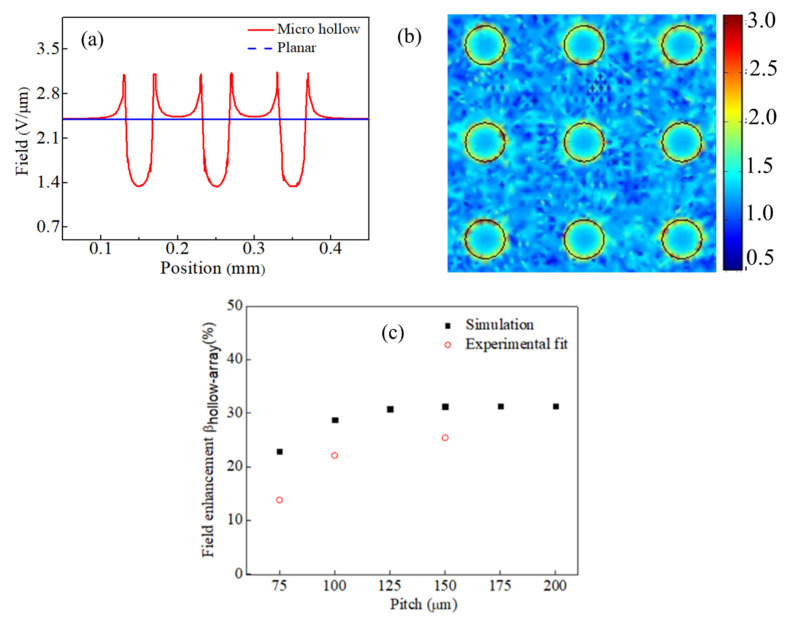
(**a**) Electric field distribution on three consecutive hollows (along centerpiece cross-section line), a maximum field at rim edges. (**b**) Contour plots of a local electric field (false color plots in V/μm scale) of 3 × 3 hollow (diameter of 40 μm, a pitch of 100 μm, depth of 200 μm), demonstrates a variation of electric field for the (**c**) simulated cathode field enhancement as a function of pitch.

Figure 3d demonstrates F–N plots of the MH-VACNTs and the planar VACNTs. In a lower field, both F–N plots were well fitted to a straight line, and linear regression analysis using the equation $\ln(I/V^{2})=a-b(1/V)$ was performed. This suggests that the observed electron emission from both cathodes follows the quantum tunnelling theory. In the high field, *F-N* plots show a saturation behavior that may be due to thermionic emission, series resistance, and cathode geometry [35,36]. The field enhancement factor β (given the known fitting value of *b* =$-B\;\phi^{1.5}d/\beta$, and assuming *Φ* = 5 eV for carbon nanotubes, ref. [37] was 4875 for the planar VACNTs. As for the MH-VACNTs (all three samples had the same 40-µm-dia. hollow, depth of ~ 200 µm), β values were 5605, 5967, and 6115 in responses to the pitch of 75, 100, and 150 μm, respectively. In other words, the local fields are increased 14%, 22%, and 25% in comparison with the planar VACNTs (Figure 4c). An increase of *β* (MH-VACNTs) is attributed to the micro hollow geometry, consistent with the simplified picture that higher emission currents appear at the vicinity of the hollow edges.

To investigate the effects of the micro hollow array on the cathode electric field, we used a 3D diode model and numerically calculated the field by using the COMSOL Multiphysics program. Cathode geometry was based on the MH-VANCT cathode; a 40 μm diameter hollow, with a depth of 200 μm, and a VANCT-layer thickness of 250 μm. Pitch *d* (hollow-to-hollow distance) is a variant in the simulation. It is noted that our model considered the cathode as a monolithic surface rather, than as porous nanotubes. In practice, to correctly calculate the electric field of the CNT cathode, it requires a meshing resolution of a few nanometers, or at least less than a nanotube diameter (~5 nm). Such resolution exceeds the number of degrees of freedom, and the available computer memory; hence, in this work we used an alternative approach to semi-quantitatively estimate field enhancement which is based on the multistage field effects [36,38]. The total field enhancement factor (β_∑_) is a product of the multistage cathode geometry (β_∑_ = β_hollow-array_ × β_CNT-film_); the simulation is to estimate β_hollow-array_. Assuming even two cathodes possessing the same β_CNT-film_, the cathode with larger β_hollow-array_ will have larger β_∑_ in general. Figure 4a,b shows the typical electric field distribution on the micro hollow with pitch *d* = 100 μm (along the centrepiece cross-section line and right on the cathode surface) and contour plots of the 3 × 3 hollow-array electric field. In the contour plots (at the X-Y plane right on the cathode surface), brighter spots are corresponding to stronger fields. In the vicinity of the hollow edge, the electric fields are strongest, and then reduce moving away from the rim edge. There was an increase of electric field moving toward the rim edge of the micro hollow, from 2.40 V/μm in the planar cathode, to a maximum of 3.09 V/μm in the vicinity of the rim edge; i.e., β_hollow-array_ = 1.28. From the SEM image of Site A in Figure 2, it can be seen that the carbon nanotube tips at the rim edge were joined together to a greater extent than site B (25 μm away from the edge). Such CNT bundles may also work as efficient emitters [39]. The laser-induced modification of carbon nanotubes, and subsequent effects on both geometry and a work function, would therefore be an interesting topic for future investigation.

A screen effect is an important factor in a cold cathode. In the case of the micro hollow cathode, too many hollows will flatten the field enhancement at the rim edge. Figure 4c plots the field enhancement (β_hollow-array_) as a function of pitch *d* in the theoretical simulation and the experimental fitting. In the numerical calculation (40-μm-dia. hollow, depth of 200 μm), the field enhancement is proportionate to *d* and saturated when *d* > 150 μm. The MH-VACNT cathode with 40 μm dia. hollow and *d* > 150 μm can enhance the field by about 31%. The field enhancement of the MH-VACNT cathodes with 40-μm-dia. hollow, and pitch d of 75, 100, and 150 μm were 23%, 29%, and 31%. These numerical calculations are reasonably consistent with the experimental data fit.

## 4. Conclusion

In this report, we presented an innovative geometry of carbon nanotubes with a micro-hollow array on the top surface, created by femtosecond laser machining. Field enhancement effects due to aspect ratios were modeled using COMSOL multiphysics. The increased geometrical field enhancement factor ultimately gives rise to the lowering of the threshold field and improved field emission current. A typical 1.5 mm dia. MH-VACNTs cathode with a hollow diameter of 40 μm, a pitch of 100 μm showed a turn-on field of 0.70 V/μm, a sustainable current of about 2 mA (~112 mA/cm^2^) at an applied field of less than 2 V/µm. Compared to the pristine and planar VACNTs, the laser-based MH-VACNT cathode can increase the local electric field by 25%. The initial characterization of VACNTs indicates that the laser process likely restructures (self-assembles) and graphitizes the carbon nanotubes, cleaning defects, and amorphous regions as well. It also improves field emission properties through factors such as geometry, work function, and stability. Finally, it can be stated the micro-hollow cathode geometry of carbon nanotubes has potential applications in high intensity, compact electron sources, and further investigation is currently underway.
